# Internet gaming disorder among individuals with autism spectrum disorder in the Kingdom of Saudi Arabia: a comparative study

**DOI:** 10.3389/fpubh.2026.1718787

**Published:** 2026-02-05

**Authors:** Mahmoud Mohamed Eltantawy, Mohammed Almutairi

**Affiliations:** Department of Special Education, College of Education, Imam Mohammad Ibn Saud Islamic University (IMSIU), Riyadh, Saudi Arabia

**Keywords:** autism spectrum disorder, behavioral addiction, comparative study, internet gaming disorder, Saudi Arabia

## Abstract

**Introduction:**

Technological advancements have produced several positive outcomes, especially for those with disabilities, as technology can help compensate for certain limitations. However, these advancements have also yielded adverse outcomes due to the overuse of technology, such as Internet gaming disorder (IGD). Therefore, this study aimed to explore the prevalence of IGD among individuals with autism spectrum disorder (ASD), determine its prevalence among males and females across different age groups, and identify differences in IGD based on sex and age.

**Methods:**

In this cross-sectional comparative study, a simple random sampling method was employed to explore the prevalence of IGD among individuals with ASD and identify differences in IGD based on sex and age. Group comparisons were conducted using the Mann–Whitney U and Kruskal–Wallis tests. This study employed the Internet Gaming Disorder Scale–Short-Form after preparing a parent-report version and verifying its psychometric properties through exploratory and confirmatory factor analyses. Overall, the study sample comprised 276 parents of children with ASD in Riyadh, Kingdom of Saudi Arabia.

**Results:**

IGD prevalence was 44.56, 49.72, and 35.35% among individuals with ASD, males, and females, respectively. Its prevalence was 27.27, 62.22, and 42.20% among individuals aged 6–12, 12–18, and >18 years, respectively. The results revealed statistically significant sex differences, with females exhibiting higher levels of IGD severity than males. Statistically significant differences were also observed across age groups, with the highest IGD levels occurring in the 12–18-year-old age group.

**Conclusion:**

IGD is prevalent among individuals with ASD, with higher rates observed during adolescence. These findings highlight the urgent need to develop targeted intervention and counseling programs, as well as provide the necessary entertainment programs and activities, to help individuals with ASD reduce their gaming time.

## 1 Introduction

Internet gaming disorder (IGD) is included in Section III of the Diagnostic and Statistical Manual of Mental Disorders, Fifth Edition, not as an officially recognized mental disorder but as a condition warranting further study (1). The International Statistical Classification of Diseases and Related Health Problems, Eleventh Revision, recognizes Gaming Disorder (GD) as a disorder resulting from addictive behaviors. GD is characterized by persistent or recurrent gaming behaviors that significantly impair personal, family, social, educational, and occupational functioning (2). Research on IGD has expanded substantially following its inclusion in these diagnostic systems (3).

IGD, one of the most common forms of Internet addiction and a primary type of behavioral addiction among adolescents (4), has emerged as a global mental health concern (5). It negatively affects individuals who engage in online gaming, influencing mental and physical well-being and reducing their quality of life (6, 7). Multiple risk factors have been associated with IGD, including anxiety, excessive playtime, family and personal difficulties, emotional problems, low self-esteem, depressive symptoms, exposure to violence, growth problems, and increased in-game purchases (8–10).

The development and persistence of IGD result from the interaction of individual, environmental, and game-related factors, rather than any single cause (11). Individual contributors include personal beliefs about game rewards, maladaptive gaming rules, reliance on gaming for social acceptance or self-esteem, and exposure to addictive triggers that alter reward processing in the brain (12, 13). Additional factors include demographic characteristics, personality traits, motivation, co-occurring mental illnesses, genetic vulnerability, and neurobiological mechanisms. Environmental influences include family dynamics, early life experiences, peer relationships, school context, cultural background, and the rising popularity of e-sports (11). Parental digital behaviors and self-efficacy also play a role in shaping gaming habits (14). Game-related features, including online structures, design elements, game types, and monetization strategies, have been shown to increase IGD risk (11). Several psychometric tools have been developed in response to increasing research interest, with the Internet Gaming Disorder Scale–Short-Form (IGDS9-SF) being one of the most widely used instruments for assessment (15).

The global prevalence of IGD varies considerably, with rates of 3.05 and 3.3% reported in a systematic review (16) and recent meta-analysis (17), respectively. Other studies have reported prevalence estimates ranging from 1 to 5% (18), whereas prevalence reached 17.0% among Chinese adolescent gamers (19). Broader international estimates range between 0.7 and 27.5%, depending on population characteristics and geographic region (20, 21). The global prevalence reached 4.6% among adolescents, with those meeting the IGD criteria exhibiting increased weekly gaming time and academic decline. Common symptoms included preoccupation and using gaming as an escape, while giving up other activities (22). Negative consequences and continued use despite problems were also identified as key diagnostic indicators (22).

Previous studies have reported a high prevalence of IGD among high school students in the Kingdom of Saudi Arabia (KSA), with rates of 21.85% among males, along with elevated depression and anxiety (23). Among females, its prevalence was estimated to be 19%, with significant positive correlations with anxiety and depression (24).

IGD appears to be common among individuals with autism spectrum disorder (ASD) (25), with studies reporting significant correlations between ASD traits and behavioral addiction (26), as well as more severe GD symptoms among individuals with ASD than among others (9). Adolescents with ASD frequently experience co-occurring mental conditions, particularly depression, which contribute to elevated rates of IGD and Internet addiction (27). The social communication challenges associated with ASD may lead to compensatory engagement in online environments, including games (28). Despite the Internet’s potential benefits for social and cognitive development, individuals with ASD are more vulnerable to problematic Internet use (29). They usually spend extended periods engaged in solitary screen-based activities, while participating less in peer interactions (30). Sensory processing differences and impulsivity, particularly among individuals with attention-deficit/hyperactivity disorder, and reinforcement from immediate digital rewards, contribute to excessive gaming (28, 31). Neurocognitive characteristics, including poor executive functioning and self-monitoring, further impair Internet use regulation (31). Adolescents with ASD usually prefer visually oriented environments to verbal communication, making online settings more comfortable and mitigating loneliness (27). Additionally, individuals with ASD frequently exhibit advanced technological abilities compared with their peers, thereby increasing their interaction with electronic devices (31).

Previous studies on IGD among individuals with ASD have reported high prevalence rates, reaching 45.45% (32) and 39.2% among adolescents with ASD (27). Differences in prevalence across studies are attributed to non-representative samples, inconsistent assessment tools, and varying conceptualizations of the disorder (18). Some studies have reported greater IGD severity among males than among females (33), whereas others have found higher levels of IGD severity and depression among females than among males (34).

Despite these findings, the diagnosis of IGD requires culturally and contextually adapted tools that are unavailable in the KSA. No reliable local data exist on IGD prevalence among individuals with ASD or on how demographic factors, such as sex and age, influence its severity. Given that IGD is shaped by individual, environmental, and game-related factors that vary across cultures, examining IGD prevalence among individuals with ASD in the KSA context is essential. However, behavioral addiction within this group remains understudied, with most studies focusing on chemical addiction (26). Moreover, reliable prevalence estimates are limited by methodological constraints, and only a few previous reviews have examined when gaming behavior becomes problematic or clinically significant (35).

Therefore, this study aimed to examine IGD among individuals with ASD by assessing its prevalence and differences in severity across sex and age groups. The research questions were as follows: What is the prevalence of IGD among individuals with ASD by sex and across age groups (6–12, 12–18, and >18 years)? Are there sex-based differences in IGD severity? and Are there age-related differences in IGD severity among individuals with ASD?

## 2 Materials and methods

### 2.1 Study design

This study employed a cross-sectional comparative design due to its ability to assess IGD prevalence and the differences in IGD severity across demographic variables, such as sex and age, at a single point in time.

### 2.2 Sample size determination

The required sample size was calculated as 257 participants using Cochran’s formula, assuming a 40% prevalence rate (*p* = 0.40), 95% confidence level, and 6% margin of error (e = 0.06). This sample size provides sufficient power (>0.80) to detect significant differences in the subsequent analytical tests. A subsequent power analysis further confirmed that this sample size was highly adequate for the planned comparative analyses—the Mann–Whitney U and Kruskal–Wallis tests.

### 2.3 Participants

Participants were recruited through an electronic questionnaire distributed to schools and institutions providing care in Riyadh, KSA. Within each participating site, eligible parents were selected using a simple random sampling method to ensure that every parent had an equal chance of being invited to participate. Although the sites were selected based on accessibility, participant selection followed random sampling principles. An electronic link to the scale was sent via WhatsApp to all parents whose children were enrolled in the participating schools or care institutions. All participating children had been previously diagnosed with ASD by accredited medical institutions, were not diagnosed for this study, and were classified as ASD level 1 (requiring support).

The sample comprised 276 parents, including 62 fathers and 214 mothers. Data were collected between February 1, 2025, and April 30, 2025. Parents of individuals with ASD were recruited as respondents for several reasons. A substantial proportion of the sampled children may have had limited reading abilities. Some information that participants were required to provide, such as engaging in the behaviors included in the scale over the past 12 months, may be difficult for certain individuals with ASD to report. Many individuals with ASD—particularly those with limited verbal skills or intellectual disabilities—may face challenges in understanding and accurately responding to questionnaires, which may affect data reliability. Direct participation can also raise ethical concerns regarding informed consent and potential distress. Individuals with ASD aged >18 years may have difficulty communicating due to limited enrollment in educational institutions. Consequently, it may be easier to communicate with one of their parents who is registered as a contact person at the school or institution where their son or daughter had studied. As primary caregivers, parents can provide reliable and accurate information about their children’s behaviors. This approach aligned with established research protocols for developmental disorders, in which proxy reporting is commonly used to ensure data validity and ethical compliance (36, 37). Table 1 presents the characteristics of the participants with ASD as described by their parents.

**Table 1:** Demographic characteristics of participants with ASD.

Variables	ASD (M/F) by age group	Frequency (n)	Percentage (%)
Sex	Number of males with ASD	177	64.1
	Number of females with ASD	99	35.9
Age Group (years)	6 ≤ age ≤12	77	27.9
	12 < age ≤18	90	32.6
	Age >18	109	39.5

ASD, autism spectrum disorder.

### 2.4 Tool

#### 2.4.1 Internet gaming disorder scale–short-form

Maldonado-Murciano et al. (15) identified the IGDS9-SF, developed by Pontes and Griffiths (38), as one of the most efficient assessment instruments for diagnosing IGD. They assessed the scale’s psychometric properties using confirmatory factor analysis (CFA), and the results supported a one-factor structure representing IGD. The nine items included in the IGDS9-SF demonstrate high validity and reliability and are appropriate for measuring IGD, thereby facilitating standardized research in this field (38).

In the Arab context, El-Keshky and Alballa (39) translated the scale and assessed its psychometric properties using Exploratory Factor Analysis (EFA) and CFA. The model fit was evaluated using Comparative Fit Index (CFI), Tucker–Lewis Index (TLI) > 0.90, Root Mean Square Error of Approximation (RMSEA), and Standardized Root Mean Square Residual <0.08, indicating an acceptable model fit. Multigroup CFA was conducted to test measurement invariance across sexes, and item response theory was applied using the multipart graded response model due to the scale’s multipart items. The EFA suggested the scale’s unidimensional structure.

##### 2.4.1.1 Justification for developing a parental version of the IGDS9-SF scale

Several previous studies have highlighted issues associated with the use of self-reports by individuals with ASD. Banker et al. (40) found that the traits self-reported by participants with ASD differed from those reported by others, raising concerns about the reliability of self-reporting. Danés and Belinchón (41) also found significant discrepancies between the self-reports of individuals with ASD and those of their parents, further questioning the reliability of self-reported data. Other scales used to diagnose GD, such as the Gaming Disorder Scale for Parents, are suitable for individuals aged 10–17 years (42), making them insufficient for this study’s objectives. The IGDS9-SF has been standardized for the Saudi population. Consequently, the researchers modified the instruments used in this study. The scale was translated from English into Arabic, reviewed by three specialists, and back-translated to confirm the accuracy of the meaning.

##### 2.4.1.2 Psychometric properties

###### 2.4.1.2.1 Content validity

Content validity was assessed through a review by five faculty members specializing in psychology and psychiatry at Saudi universities. They evaluated the revised version of the IGDS9-SF Parenting Scale for conceptual consistency, cultural appropriateness, and linguistic clarity. This process resulted in modifications to some of the scale items, including the simplification of certain terms.

###### 2.4.1.2.2 Pilot testing

A pilot test was conducted with 22 parents of children with ASD to assess the scale’s reliability and clarity. This initial test provided preliminary feedback on the scale’s effectiveness in capturing relevant data and helped identify and refine ambiguous vocabulary.

###### 2.4.1.2.3 Exploratory factor analysis

The IGDS9-SF scale was applied after completing content validation and pilot testing, and EFA was performed on the nine-item scale. Principal pivotal factor analysis was used to extract the principal factors. The sample fit was confirmed with a value of 0.659 using the Kaiser–Meyer–Olkin coefficient, exceeding the recommended minimum of 0.60 (43). Bartlett’s test for sphericity was statistically significant (*p* < 0.001), indicating that the correlation matrix was suitable for factor analysis. EFA extracted only a single factor with eigenvalues greater than one, explaining 52.691% of the total variance. All items had factor loads >0.40 (44).

###### 2.4.1.2.4 Confirmatory factor analysis and reliability

Following the EFA, which confirmed the scale’s unistructural nature, CFA was conducted. The results revealed that the proposed measurement model has a good fit to the data, with the quality of fit indices falling within acceptable limits (CFI = 0.916, TLI = 0.939, and RMSEA = 0.073) (45), indicating the validity of the scale’s factor construct. Factor loading coefficients ranged between 0.714 and 0.857, all of which are statistically significant, confirming the items’ consistency (46). The construct validity and reliability indices also showed high values, with Composite Reliability and Average Variance Extracted of 0.715 and 0.721, respectively, reflecting the instrument’s high construct validity and reliability (47). The scale’s internal reliability was calculated using the alpha coefficient. Cronbach’s alpha, with a value of *α* = 0.714, is considered acceptable (48).

In the original version of the IGDS9-SF scale developed by Pontes and Griffiths (38), the values were CFI = 0.964, TLI = 0.952, and RMSEA = 0.054, with a Cronbach’s alpha coefficient of α = 0.87. Comparing the original scale with the version adapted for parents in the Arab context revealed slight differences in the fit indices and Cronbach’s alpha. This slight difference is attributed to several factors, including the smaller sample size in the current study, cultural specificities in the Arab context, such as the social stigma associated with acknowledging IGD, linguistic challenges in translating some items, and the adaptation of the version to suit parents. Such variations are common in cultural adaptation processes and do not indicate a weakness in the scale’s validity (49).

###### 2.4.1.2.5 Score calculation method

The final version of the assessment tool comprised two sections. The first section was a survey designed to collect general information, including participants’ sex, age, consent for the use of the information provided by parents for this study, and confirmation that the behaviors listed on the scale occurred within the past 12 months. In contrast, the second section comprised the scale, which included a set of specific items representing the behaviors being measured.

The scale consists of nine items. Participants responded to each item by selecting from alternatives (1–5). The scale’s scores ranged between 9 and 45 points. A five-point scale was used (1 = never, 2 = rarely, 3 = sometimes, 4 = often, and 5 = very often), with a higher score indicating a higher degree of IGD. To differentiate individuals with IGD, the participants had to agree with at least five out of nine criteria, by considering answers such as “5 = Very Often,” which translated as endorsement of the criterion (Appendix).

### 2.5 Data collection and analysis

School and care institution administrators were contacted to distribute the scale to all parents using WhatsApp after obtaining approval from the Institutional Ethics Committee of Imam Mohammad ibn Saud University. The scale was electronically administered using Google Forms. They were also informed of the study’s objectives and procedures, and informed consent was obtained to use the data. The participants were informed that their data would be handled and stored confidentially, ensuring that no information would be disclosed. Moreover, they were informed that their participation was entirely voluntary and that they could withdraw at any time without consequences.

Data were analyzed using IBM SPSS Statistics for Windows, version 27 (IBM Corp., Armonk, N. Y., United States) and SmartPLS (version 4.1.1.6). The scale’s reliability was assessed using Cronbach’s alpha, with a threshold of ≥0.70 considered acceptable. Normality of the score distribution was assessed using Kolmogorov–Smirnov and Shapiro–Wilk tests, with significant results (*p* < 0.05) indicating a non-normal distribution. Levene’s test was conducted to assess the homogeneity of variances, and the results indicated that variance homogeneity was achieved. The Mann–Whitney U test was used to compare two independent groups and perform post-test comparisons. Multiple groups comparison was performed using the Kruskal–Wallis test.

## 3 Results

IGD prevalence percentages were calculated for the entire study sample, comprising 276 individuals with ASD, to answer the first research question. Subsequently, IGD prevalence percentages were analyzed according to sex and age. Table 2 presents the responses to this question. As shown in Table 2, a total of 123 individuals with ASD met the IGD criteria (i.e., scoring 5 on five or more items), representing 44.56% of the sample. Of these, 88 (49.72%) and 35 (35.35%) were males and females, respectively. According to age group, 21 (27.27%), 56 (62.22%), and 46 (42.20%) children were aged 6–12, 12–18, and >18 years, respectively.

**Table 2:** IGD prevalence among individuals with ASD according to specific variables.

Variables	Category	Number of individuals with IGD	Total number	%
Prevalence	Individuals with ASD and IGD	123	276	44.56
Prevalence according to sex	Males	88	177	49.72
	Females	35	99	35.35
Prevalence according to age group	6 ≤ age ≤12	21	77	27.27
	12 < age ≤18	56	90	62.22
	Age >18	46	109	42.20

ASD, autism spectrum disorder; IGD, Internet gaming disorder.

The Mann–Whitney U test was used to address the second research question, regarding the differences in IGD severity among individuals with ASD based on sex. Table 3 presents the responses to this question. The Z value was −7.020, and the significance level was 0.001, indicating statistically significant differences, with females exhibiting higher levels of IGD severity than males (Table 3).

**Table 3:** Mann–Whitney test results for differences between groups according to sex.

Scale	Sex	N	Mean rank	Sum of ranks	Z	Sig
IGD	Male	177	113.35	20062.50	−7.020	0.001
	Female	99	183.47	18163.50		

Sig, significance level; IGD, Internet gaming disorder.

The third research question, examining the differences in IGD severity among individuals with ASD based on age, was addressed using the Kruskal–Wallis test. Table 4 presents the responses to this question. As shown in Table 4, the significance level was 0.027, indicating statistically significant differences at the 0.05 significance level among the mean scores of the three groups on the IGD scale.

**Table 4:** Kruskal–Wallis test for differences between groups according to the age group.

Scale	Age group (years)	N	Mean rank	Value of the Kruskal–Wallis test	Sig
IGD	6 ≤ age ≤12	77	131.75	7.188	0.027
	12 < age ≤18	90	156.84		
	Age >18	109	128.12		
	Total	276			

Sig, significance level; IGD, Internet gaming disorder.

To determine the direction of the differences among the three groups, each pair of groups was compared using the Mann–Whitney U test. Table 5 presents the responses to this question. As shown in Table 5, statistically significant differences were observed at the 0.05 significance level between the mean scores of the 6–12- and 12–18-year-old groups, with higher scores in the 12–18-year-old group; statistically significant differences were observed between the mean scores of the 12–18- and >18-year-old groups, with higher scores in the 12–18-year-old group; and no statistically significant differences were observed between the 6–12- and >18-year-old groups.

**Table 5:** Mann–Whitney test results for differences between groups according to the age group.

Age group (years)	6 ≤ age ≤12	12 < age ≤18	Age >18
6 ≤ age ≤12	_____	−2.030*	−0.307
12 < age ≤18		_____	−2.531*
Age >18			_____

*indicates statistical significance at *p* < 0.05.

## 4 Discussion

The findings revealed that IGD prevalence among individuals with ASD in KSA was remarkably high, consistent with previous studies, including Simonelli et al. (32), who reported a 45.45% prevalence among individuals with ASD, and Tateno et al. (27), who reported a 39.2% prevalence among adolescents with ASD. This high prevalence can be traced back to the statistically significant positive correlation between behavioral addiction and ASD (26) and the presence of multiple co-occurring disorders in individuals with ASD, one of which is IGD (27). Some studies equated this high prevalence with the social issues associated with ASD, which may drive individuals toward the compulsive use of Internet games to compensate for the skills they lack (28). The traits and characteristics of individuals with ASD, such as different sensory processing leading to a tendency toward visual content, have been reported to contribute to excessive gaming (28). Notably, the rapid digital expansion in KSA may have contributed to the increased prevalence of IGD, given that these rates were considerably higher than those reported in other countries. One possible explanation for this is the country’s high economic status, which ensures widespread access to electronic devices and the financial capacity to purchase digital games. Supporting this, the Saudi Internet Report indicated that Internet penetration in the KSA has reached 99% of the population (50).

The findings corroborate that IGD prevalence was higher among males than among females, consistent with the results of Labrador et al. (33) and Murray et al. (25), confirming a higher IGD prevalence among males than among females. According to age, IGD prevalence among individuals with ASD was the highest in the 12–18-year-old age group, lower in the >18-year-old age group, and the lowest in the 6–12-year-old age group. These findings align with those of several studies reporting an elevated prevalence of IGD among adolescents (19, 23). This may be attributed to the nature of this stage, during which individuals make friends and establish social relationships with others, which is a challenge that individuals with ASD may fail to overcome, leading them to seek alternative activities. This was consistent with the findings of Shane-Simpson et al. (28).

Regarding the differences in IGD severity among individuals with ASD based on sex, the findings demonstrated that females exhibited higher levels of IGD severity than males. These were consistent with the findings of Minami et al. (34), who reported that females experienced the disorder more severely than males. Similarly, Wang et al. (51) confirmed that IGD impacts female brain structure more severely than male brain structure. However, these findings diverge from those of Gisbert-Pérez et al. (52), who reported statistically significant differences in weekly gaming, with males spending more time gaming. They also contradicted the findings of Zhang et al. (53), who revealed that male gamers were more sensitive to game rewards and less sensitive to losses than females, potentially justifying the discrepancy in-game participation rates between the two sexes. Dong and Potenzae (54) suggested that this can be attributed to the fact that IGD is linked to several cultural, social, and economic variables, the most important of which are socialization and cultural barriers (55). This is evident in Saudi society, where few recreational opportunities exist for females and obstacles that limit the practice of physical activities are prevalent, such as a lack of services and social and family obstacles (56). The motivations of females to participate in recreational activities are linked to cultural factors, such as the availability of safe places to practice recreation and previous negative experiences (57). Additionally, families of individuals with ASD in Saudi society face many challenges at the psychological, social, emotional, and financial levels (58), given that 33.7% of parents of individuals with ASD experience stigma, with mothers reporting more self-stigma than fathers, which may affect the provision of recreational services for individuals with ASD (59). Similarly, the high level of overprotection among young females in the KSA may also contribute to these outcomes (60).

Regarding differences in IGD severity among individuals with ASD based on age, the findings indicated that severity was highest in the 12–18-year-old group. Notably, this developmental stage represents adolescence. Several studies have reported that interest in Internet gaming peaks during adolescence (19, 23, 28). Adolescents are more prone to IGD because they spend a long time playing these games (61, 62).

Particularly, the characteristics of ASD interact with those of adolescence, making adolescents feel torn between two forces pulling in different directions: the necessity for social interaction and establishing friendships, and ASD with its various symptoms, including social interaction deficit, which forces adolescents to search for a middle ground (i.e., getting immersed in gaming). Moreover, parental control weakens with age, particularly during adolescence (63). The decline in IGD severity among individuals aged >18 years can be traced back to this stage being one during which individuals typically have a job or are enrolled in university, resulting in having less time for gaming. Similarly, the decline in IGD severity among children aged 6–12 years, compared with the other two age groups, can be justified by children of this age usually being busy playing with their peers. Alfaifi et al. (64) emphasized the role played by environmental factors in shaping IGD. For example, a lack of available entertainment venues can leave adolescents with no alternative but to spend more time at home, where they are most vulnerable to IGD.

Notably, this study has limitations that must be considered when generalizing its findings. First, it relied on parental diagnoses of IGD, which may have introduced observer bias. Parents might have misinterpreted the internalizing behaviors of individuals with ASD (such as preoccupation) as gaming addiction, when, in fact, they may have restricted interests associated with ASD. Second, given that this was a cross-sectional study, it cannot be claimed that playing online games causes problems. Finally, the study was conducted in Riyadh, an urban city, and the results indicated a higher prevalence of IGD in urban areas than in rural areas (65), thereby limiting the generalizability of the findings to other populations.

## 5 Conclusion

This study explored the prevalence of IGD among individuals with ASD, examining prevalence rates by sex and across different age groups, as well as differences in IGD severity based on sex and age. Certain ministries and institutions, such as the Ministry of Education and Ministry of Health, can implement screening programs for middle and high school students to identify IGD, conduct workshops to distinguish between passive (flow) and pathological (addiction) gaming, and provide entertainment and sports activities for individuals with ASD. This study recommends conducting additional studies on IGD on large samples and experimenting with different environments in terms of cultural backgrounds. Additionally, studies should be conducted to diagnose the disorder based on the relationship between a parent and an individual with ASD. Finally, while gaming offers a digital refuge for Saudi youth with ASD without regulation, it risks becoming a pathological trap, necessitating culturally sensitive, family-centered interventions.

## Data Availability

The data analyzed in this study is subject to the following licenses/restrictions: the datasets generated and analyzed during the current study are available from the corresponding author upon request. Requests to access these datasets should be directed to Mahmoud Mohamed Eltantawy Department of Special Education, College of Education, Imam Mohammad Ibn Saud Islamic University (IMSIU), Saudi Arabia.

## Appendix

The Parent Version of the Internet Gaming Disorder Scale – Short Form (IGDS9-SF):

Instructions: These questions ask you about your son’s or daughter’s gaming activity during the past year (i.e., the last 12 months). By gaming activities, we refer to any gaming-related activity that has been played either using a computer/laptop, gaming console, or any other kind of device (e.g., mobile phone, tablet, etc.), both online and offline.

**Table:**

Item	Statement	Never	Rarely	Sometimes	Often	Very often
1	Does your son/daughter appear preoccupied with gaming? For example: thinking about games even when not playing, planning the next gaming session, or perceiving gaming as the dominant daily activity.	◯	◯	◯	◯	◯
2	Does your son/daughter seem to experience increased irritability, anxiety, or even sadness when you try to reduce or stop their gaming activity?	◯	◯	◯	◯	◯
3	Does your son/daughter appear to need to spend increasing amounts of time gaming to achieve satisfaction or pleasure?	◯	◯	◯	◯	◯
4	Does your son/daughter consistently fail in attempts to control or stop their gaming activity?	◯	◯	◯	◯	◯
5	Has your son/daughter lost interest in previous hobbies and other leisure activities as a result of excessive gaming?	◯	◯	◯	◯	◯
6	Does your son/daughter continue gaming despite being aware of the problems it causes with others?	◯	◯	◯	◯	◯
7	Has your son/daughter deceived family members, therapists, or others regarding the amount of time spent gaming?	◯	◯	◯	◯	◯
8	Does your son/daughter play games to temporarily escape or relieve negative moods (e.g., helplessness, guilt, anxiety)?	◯	◯	◯	◯	◯
9	Has your son/daughter jeopardized or lost an important relationship, job, educational opportunity, or career prospect owing to gaming?	◯	◯	◯	◯	◯
